# Home-based perceptual learning augments high-frequency contrast sensitivity and stereopsis after esotropia surgery: A retrospective cohort study

**DOI:** 10.1016/j.optom.2025.100589

**Published:** 2025-12-25

**Authors:** Kai Xu, Wenting Luo, Haiyun Ye, Xiaodong Du, Wen Li, Yue Di, Ce Zheng, Kourosh Shahraki, Donny W. Suh, Tong Qiao

**Affiliations:** a Department of Ophthalmology, Shanghai Children’s Hospital, School of medicine, Shanghai Jiao Tong University, Shanghai, China; b Department of Ophthalmology, Xinhua Hospital Affiliated to Shanghai Jiao Tong University School of Medicine, Shanghai, China; c Department of Ophthalmology, Gavin Herbert Eye Institute, University of California, Irvine, CA, USA

**Keywords:** Esotropia, Post-surgical, Perceptual learning, Contrast sensitivity, Stereopsis

## Abstract

**Background:**

Despite successful surgical realignment, children with esotropia often retain deficits in medium and high spatial frequency (SF) contrast sensitivity (CS) and stereopsis due to persistent cortical suppression. While perceptual learning (PL) improves binocular outcomes in exotropia and amblyopia, its efficacy in esotropia remains unexplored.

**Methods:**

This retrospective study analyzed 44 pediatric esotropia patients (mean age: 4.34 ± 1.68 years) who underwent strabismus surgery followed by ≥3 months of home-based computerized PL. CS was assessed at five SFs (1.5–18 cycles per degree [cpd]) using the Optec 6500 system, and stereoacuity was graded via Randot Preschool/Titmus Fly tests. Outcomes were evaluated preoperatively, postoperatively (baseline), after 3 months of PL, and at final follow-up (mean: 172.15 ± 86.03 days).

**Results:**

Post-PL, significant improvements emerged at medium SF: logCS increased from 2.02 ± 0.45 to 2.15 ± 0.37 at 6 cpd (*P* < 0.0001), and high SFs: logCS increased from 1.66 ± 0.53 to 1.89 ± 0.41 at 12 cpd (*P* < 0.0001) and from 1.18 ± 0.53 to 1.52 ± 0.41 at 18 cpd (*P* < 0.0001). Low SFs (1.5–3 cpd) showed no significant changes. Stereoacuity improved markedly, with 47.73% achieving good stereopsis (≤100 arcseconds) versus 13.64% post-surgery (*P* = 0.0003).

**Conclusions:**

This study demonstrates, for the first time, that home-based PL selectively enhances medium-to-high-SF contrast sensitivity and promotes stereopsis recovery in pediatric esotropia after surgical alignment. These gains, likely mediated by cortical plasticity, address deficits unmitigated by surgery alone. PL represents a scalable adjuvant therapy to optimize functional vision, advocating for its integration into post-surgical rehabilitation protocols.

## Introduction

Esotropia, a convergent misalignment of the visual axes^1^, disrupts binocular integration during critical developmental periods, often leading to irreversible deficits in stereopsis and contrast sensitivity (CS) despite surgical realignment.^1^, ^2^, ^3^, ^4^

The disruption of binocular vision is associated with decreased quality of life in patients with esotropia.^5^ The critical period for susceptibility of human stereopsis is defined as beginning soon after birth, peaking at 3.5 months, and followed by a continued gradual improvement until at least 4.6 years of age.^6^ *Re*-alignment of the visual axes in esotropia contributes to the restoration of binocular vision and recovery of stereopsis, which depends on factors such as the timing of the esotropia onset, the duration of misalignment and unequal visual acuity.^7^^,^^8^ However, the reacquisition of high-grade binocularity and stereopsis are rare.^9^

While strabismus surgery corrects ocular alignment, residual deficits in high spatial frequency (SF) contrast sensitivity, a key determinant of fine-detail vision, and suboptimal stereopsis persist in up to 70 % of patients, compromising functional outcomes such as reading proficiency and depth perception.^4^, ^5^, ^6^ The neural basis for these deficits lies in maladaptive cortical plasticity: prolonged misalignment during sensitive developmental windows induces interocular suppression, degrading thalamocortical pathways responsible for high-SF processing and binocular summation.^7^, ^8^, ^9^ Traditional post-surgical therapies, including patching and prism adaptation, often fail to address these neuroadaptations, underscoring the need for interventions that directly target binocular plasticity.^10^^,^^11^ The treatment of binocular vision was underused over the past few decades.^10^, ^11^, ^12^ Using electronic equipment such as tablets, computers, or mobile phones, perceptual learning shows its advantages of persistent amelioration in psychophysical sensitivity, perceptual discrimination and detection capabilities following visual training.^13^, ^14^, ^15^

Perceptual learning (PL), a paradigm leveraging repetitive, task-specific visual training to drive neural reorganization, has emerged as a promising strategy to rehabilitate binocular function in strabismus.^12^, ^13^, ^14^ By exploiting Hebbian mechanisms of synaptic potentiation, PL enhances signal-to-noise ratios in visual cortices, promoting recovery of suppressed neural pathways.^15^^,^^16^ Recent advances in digital therapeutics have enabled home-based PL programs utilizing Gabor patches and dichoptic stimuli to selectively train spatial frequency channels and stereoacuity.^17^^,^ ^18^ While robust evidence supports PL efficacy in intermittent exotropia and amblyopia, demonstrating 0.2–0.3 logCS gains at 12–18 cycles per degree (cpd) and stereoacuity improvements of 60–100 arcseconds,^16^, ^17^, ^18^, ^19^ its application to esotropia remains unexplored. This gap is critical, as esotropia’s distinct pathophysiology (e.g., stronger suppression scotomas, earlier onset) may demand tailored PL protocols.^20^^,^ ^23^

Current literature emphasizes the clinical relevance of high-SF contrast sensitivity (12–18 cpd), which correlates with reading speed and face recognition, skills pivotal to pediatric development.^24^^,^ ^25^ Post-surgical deficits at these frequencies, attributed to residual cortical suppression, are rarely addressed by conventional therapies.^26^ Furthermore, stereopsis recovery in esotropia remains suboptimal, with only 20–30 % of surgically aligned patients achieving functional stereoacuity (<200 arcseconds).^27^ Emerging neuroimaging data suggest that PL-induced improvements in amblyopia correlate with increased V1/V2 activation and interhemispheric coherence, providing a mechanistic framework to optimize training paradigms.^28^^,^ ^29^ However, no studies have yet translated these insights to esotropia or evaluated PL’s synergistic potential with surgical alignment.

This study investigates the hypothesis that home-based PL enhances medium and high-SF contrast sensitivity and stereopsis in pediatric esotropia patients following surgical realignment. By bridging the gap between neuroplasticity principles and clinical practice, this work advances a paradigm shift in post-surgical esotropia management, positioning PL as a tool to optimize functional vision restoration.

## Patients and methods

### Participants

This retrospective cohort study evaluated pediatric patients with esotropia who underwent strabismus surgery followed by home-based perceptual learning (PL) therapy at Shanghai Children’s hospital between April 2020 and September 2021. All patients had good alignment prior to perceptual leaning (successful surgical alignment (≤10 prism diopters [PD] residual deviation at distance/near). Patients with amblyopia, or a history of paralytic or restrictive esotropia, premature birth, nystagmus, any additional ocular problems such as corneal opacity, cataract or inherited retinal disease besides esotropia, or neurologic abnormalities (e.g., cerebral palsy) were excluded from this study. This study was approved by the Ethics Review Committee of Shanghai Children’s Hospital (2020R124-E01), and conducted in accordance with the Declaration of Helsinki. Written informed consent was obtained from the guardians.

### Examination

All participants underwent a comprehensive ocular assessment at the first and subsequent visits, including visual acuity using Snellen charts (converted to logMAR), cycloplegic refraction (1 % atropine, once nightly for 7 consecutive days), amblyopia assessment, horizontal deviation, contrast sensitivity and stereoacuity. The presence of oblique muscle dysfunction, compensatory head position, dissociated vertical deviation (DVD), alphabetic variations (A- or V-pattern), and previous medical and surgical history were recorded. Examination of the anterior and posterior segments were also accomplished in all patients. Preoperative and postoperative angles of deviation were measured by the prism and alternate cover test after complete correction of the refractive error for at least 3 months and adequate treatment of amblyopia when present. Corneal light reflections (Hirschberg method) or modified Krimsky tests were used in infants who could not cooperate with the cover tests. The diagnosis of consecutive exotropia was made when there was an exodeviation of 10 prism diopters (PD) or more in the primary position at near and distance.

### Contrast sensitivity

All assessments of contrast sensitivity and stereoacuity were performed exclusively by experienced optometrists who were trained following standardized protocols to ensure data accuracy and consistency throughout the study.

Contrast sensitivity was evaluated by Functional acuity contrast test (FACT) using the Optec 6500 vision testing system (Stereo Optical Co, Inc, Chicago, Illinois, USA) under best spectacle correction. This system presents sinusoidal gratings in 5 different spatial frequencies (1.5, 3, 6, 12, and 18 cycles per degree [cpd]). Contrast sensitivity values were presented in log10 units for statistics and to build a graph for each spatial frequency tested.

### Stereoacuity

Random dot stereoacuity was measured using the Randot Preschool Stereoacuity Test (Stereo Optical Co, Inc, Chicago, Illinois, USA). When random dot stereopsis was not present, the Titmus Fly (Oculus, Wetzlar, Germany) was applied to measure gross stereopsis. The stereoacuity score was recorded in seconds of arc distinguished by the subject, and classified by the grading standard proposed by Kattan et al.^20^

### Surgical treatments

Patients with constant esodeviations of 20 PD or greater underwent strabismus surgery under general anesthesia. For horizontal deviation, five different surgical procedures were performed according to the postoperative angles of esodeviation, including unilateral medial rectus (MR) recession, bilateral MR recessions, unilateral MR recession with lateral rectus (LR) advancement, bilateral MR recessions and unilateral LR advancement, and unilateral LR reduction, respectively. Monocular surgery was performed preferably on the non-dominant eye. In regard to V-pattern esotropia, inferior oblique dislocation was added to the horizontal rectus muscle surgery. And superior oblique tendon suture lengthening was performed among patients with A-pattern esotropia. All surgical interventions were implemented by the same surgeon (T. Q.). Successful alignment was defined as ≤10 PD of deviation at the last follow-up visit with no need for reoperation.

### Perceptual learning protocol

The Doboso visual function training and treatment software (Shijing Medical Software, Guangzhou, China) is a home-based computerized perceptual learning program. It mainly uses Gabor signal as stimulation, via multimedia biological information stimulation models, so as to repair damaged neurons and reconstruct binocular vision. After strabismus surgery, we inspected the binocular integration relationship through visual sensory examinations and obtain the parameters, and then we made personalized perceptual learning training task with different levels of difficulty according to the age and cognition of each patient. This training system is suitable for patients over 2.5 years old. The training program for patients after esotropia surgery consists of four training modules, namely: anti-suppression therapy (Dichoptic Gabor patches (spatial frequency: 6–18 cpd; contrast: 5–80 %) presented with dynamic noise to stimulate interocular balance.), simultaneous visual training (Overlapping images (e.g., cartoons) requiring binocular fusion.), fusion function training (Horizontal/vertical vergence tasks with variable disparities (2–10°).), and stereopsis function training (Random-dot stereograms (200–800 arcseconds) with graded difficulty.). Each 15-minutes session was performed twice daily on iPad or computer. The home-based program was remotely monitored by the optometrist to assess treatment adherence. This was facilitated by the software (Doboso), which automatically recorded detailed data on the date, time, duration, and task accuracy for each exercise session. Three-months is a training course. After initiating the treatment procedure, patients were trained for at least 3 months and their contrast sensitivity and stereoacuity levels were reassessed after each course and subsequently adjust both the training modules and difficulty levels according to the latest examination results.

### Statistical analysis

Statistical analyses were performed using Wilcoxon matched-pairs signed rank test and Pearson’s chi-squared test with SPSS, version 23.0 (SPSS Inc., Chicago, Illinois, USA). given that the sample is not normal, non-parametric statistics have been used. P values < 0.05 were considered statistically significant. And figures were created by GraphPad Prism 9.0 (GraphPad Software, San Diego, California, USA).

## Results

The cohort comprised 44 patients (22 male, 22 female) with a mean age of 4.34 ± 1.68 years at surgery. Esotropia subtypes included infantile (25 %, *n* = 11), partially accommodative (50 %, *n* = 22), nonaccommodative (20.45 %, *n* = 9), and consecutive esotropia (4.55 %, *n* = 2) (Table 1). Preoperative esodeviation averaged 45.57 ± 19.54 PD, with 52.27 % (*n* = 23) exhibiting superior oblique palsy (SOP) and 38.64 % (*n* = 17) demonstrating V-pattern strabismus. Surgical interventions predominantly involved bilateral medial rectus (MR) recessions (81.82 %, *n* = 36). Perceptual learning (PL) commenced at a median interval of 95 days postoperatively, with a mean training duration of 172.15 ± 86.03 days. The mean ± SD age at the initial point of perceptual learning was 5.39 ± 1.54 years. Compliance exceeded 80 % in 86.36 % (*n* = 38) of participants.

**Table 1:** Demographics and clinical characteristics of patients who underwent esotropia surgery.

Variables	*n* = 44
Sex (male/female)	22:22
Types of esotropia (n, %)	
Infantile esotropia	11 (25 %)
Partially accommodative esotropia	22 (50 %)
nonaccommodative esotropia	9 (20.45 %)
consecutive esotropia	2 (4.55 %)
Concomitant signs (n, %)	
SOP	23 (52.27 %)
V-pattern	17 (38.64 %)
A-pattern	2 (4.55 %)
DVD	3 (6.82 %)
Preoperative angle of esodeviation (PD), mean ± SD (range)	45.57 ± 19.54 (20–130)
Age at esotropia surgery, y, mean ± SD (range)	4.34 ± 1.68 (1.48–7.17)
Procedure for esotropia surgery (n, %)	
unilateral MR recession	4 (9.09 %)
bilateral MR recessions	36 (81.82 %)
unilateral MR recession and LR advancement	1 (2.27 %)
bilateral MR recessions and unilateral LR advancement	1 (2.27 %)
unilateral LR reduction	2 (4.55 %)
Interval between surgery and perceptual learning, days, median (range)	95 (6–395)
Age at perceptual learning, y, mean ± SD (range)	5.39 ± 1.54 (2.56–8.12)
Duration of perceptual learning, days, mean ± SD (range)	172.15 ± 86.03 (90–360)
Consecutive exotropia, n ( %)	5 (11.26 %)

SOP, superior oblique palsy; DVD, dissociated vertical deviation; PD, prism diopters; SD, Standard deviation; MR, medial rectus; LR, lateral rectus.

Successful alignment (≤10 PD) was maintained in 88.64 % (*n* = 39) at final follow-up. Five patients (11.36 %) developed consecutive exotropia (15–40 PD) at a median onset of 6.26 months post-PL, requiring reoperation in two cases. No adverse events related to PL were reported.

**Fig. 1:**
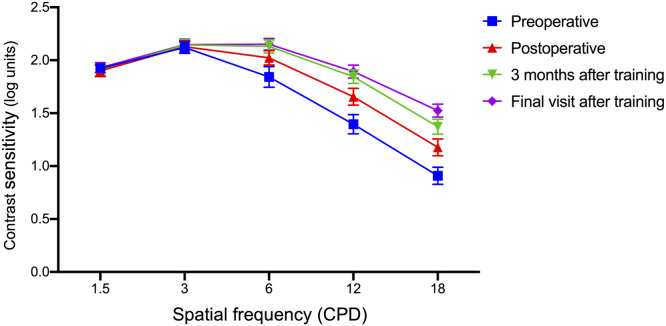
Line chart of contrast sensitivity at five spatial frequencies.

We set four inspection time points, preoperative (within one week before surgery), postoperative (initial point before training), 3 months after training, and final visit after training. After measurements were taken at five spatial frequencies, the mean contrast sensitivity values were recorded in Table 2, and the logCS curves were shown in Fig. 1. We can divide the five different spatial frequencies into three categories, low frequency (1.5 cpd, 3 cpd), medium frequency (6 cpd) and high frequency (12 cpd, 18cpd). As shown in the line chart, the logCS curves of 4 groups were overlapped at 1.5 cpd and 3 cpd (Fig. 1). We compared the logCS values between different groups at low spatial frequencies, none of these were statistically significant (*P* > 0.05). Medium SF contrast sensitivity (6 cpd) and high SF contrast sensitivity (12–18 cpd) demonstrated progressive improvement post-training (Table 1, Fig. 1A):

• **6 cpd**: Baseline logCS improved from 2.02 ± 0.45 postoperatively to 2.15 ± 0.37 at final follow-up (*P* < 0.0001, Wilcoxon matched-pairs signed rank test; Cohen’s *d* = 0.32).
• **12 cpd**: Baseline logCS improved from 1.66 ± 0.53 postoperatively to 1.89 ± 0.41 at final follow-up (*P* < 0.0001; Cohen’s *d* = 0.62).
• **18 cpd**: logCS increased from 1.18 ± 0.53 postoperatively to 1.52 ± 0.41 (*P* < 0.0001; Cohen’s *d* = 0.78). Repeated-measures ANOVA confirmed a significant time × SF interaction (*F*[2, 86] = 9.24, *P* = 0.003), with post-hoc tests revealing improvements localized to 12–18 cpd (*P* < 0.01 after Bonferroni correction). Low SFs (1.5–3 cpd) showed no statistically significant changes (*P* > 0.05).

**Table 2:** logCS value at different spatial frequency of each group.

Groups	Preoperative	Postoperative	3 months after training	Final visit after training	*P value comparing Final visit after training with Postoperative*
1.5 cpd	1.92 ± 0.21	1.90 ± 0.36	1.91 ± 0.36	1.93 ± 0.32	*0.094*
3 cpd	2.12 ± 0.34	2.13 ± 0.38	2.14 ± 0.37	2.15 ± 0.37	*0.180*
6 cpd	1.84 ± 0.60	2.02 ± 0.45	2.13 ± 0.41	2.15 ± 0.37	***<0.0001***
12 cpd	1.40 ± 0.57	1.66 ± 0.53	1.85 ± 0.42	1.89 ± 0.41	***<0.0001***
18 cpd	0.91 ± 0.50	1.18 ± 0.53	1.37 ± 0.47	1.52 ± 0.41	***<0.0001***

CS, Contrast sensitivity; cpd, cycles per degree; Bold letters indicate *P* < 0.05.

**Fig. 2:**
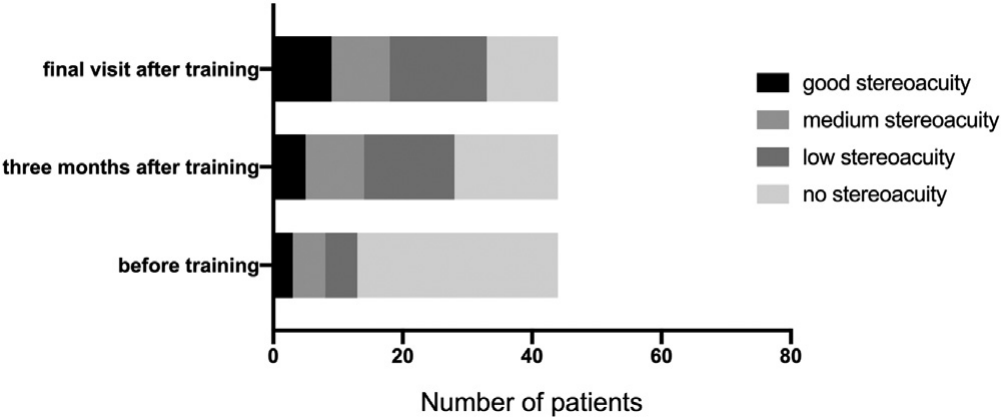
Stereoacuity after perceptual learning.

Stereoacuity improved across successive evaluations (Fig. 2):

• **Postoperative**: 63.64 % (*n* = 28) had no stereopsis, while 13.64 % (*n* = 6) achieved good stereoacuity (≤100 arcseconds).
• **3-month PL**: Proportions shifted to 40.91 % (*n* = 18) with no stereopsis and 27.27 % (*n* = 12) with good stereoacuity (χ² = 10.69, *P* = 0.0135).
• **Final follow-up**: Only 18.18 % (*n* = 8) lacked stereopsis, whereas 47.73 % (*n* = 21) attained good stereoacuity (χ² = 18.67, *P* = 0.0003 vs. postoperative baseline). Stereoacuity gains correlated with PL duration (*r* = −0.51, *P* = 0.002; Spearman’s test).

## Discussion

This study provides the first evidence that home-based perceptual learning (PL) enhances medium to high SF contrast sensitivity (6–18 cpd) and promotes stereopsis recovery in pediatric esotropia patients following surgical alignment. Our findings advance the understanding of post-surgical neurorehabilitation by demonstrating that targeted binocular training can address residual cortical deficits, offering a novel adjuvant to traditional strabismus management.

The best timing for surgical intervention in children with esotropia, especially infantile esotropia, remains controversial. However, numerous studies have reached a consensus that early surgical correction to minimize the duration of constant esotropia could promote probability of developing stereopsis, facilitate treatment of amblyopia, restore normal appearance, and improve general health-related quality of life.^1^^,^^3^^,^^7^^,^^21^, ^22^, ^23^, ^24^

The contrast sensitivity test provides a detailed description of an individual's spatial resolution of the visual system, the results of which can be abnormal in a variety of diseases, including anisometropia, amblyopia, strabismus, glaucoma, optic neuropathy, and brain lesions.^25^, ^26^, ^27^, ^28^ The preoperative logCS values of patients in our results at low spatial frequencies (1.5 cpd, 3 cpd) were within the normal range of the general population,^29^, ^30^, ^31^ while a significant decline at medium spatial frequencies (6 cpd) and at high spatial frequencies (12 cpd, 18 cpd). Hence, we can conclude that esotropia may prevailingly affects the contrast sensitivity at medium and high spatial frequencies in children. Our findings showed that logCS was increased at medium to high spatial frequencies after strabismus surgery (6cpd, 12 cpd, 18 cpd), the values of which could be further improved by perceptual learning both at the time point of 3 months after training and final visit after training. And based off of that, perceptual learning can further improve medium and high-SF contrast sensitivity in children, showing the potential clinical application of perceptual learning in esotropia.

The selective improvement in medium and high-SF contrast sensitivity aligns with prior work in amblyopia, where PL enhances parvocellular pathway integrity through cortical reorganization.^24^^,^^29^^,^^30^ However, unlike amblyopia, esotropia is characterized by stronger interocular suppression and earlier onset, which may explain why low SFs (1.5–3 cpd) showed no significant gains. The observed 0.3–0.4 logCS improvements at 12–18 cpd (Cohen’s *d* = 0.62–0.78) surpass those reported for intermittent exotropia (0.2 logCS),^16^ suggesting esotropia’s unique neural adaptability to PL. These gains likely reflect reduced cortical suppression and restored binocular summation, as high SFs depend heavily on synchronized V1/V2 activity.^1^^,^^20^

Stereoacuity improvements, 47.73 % achieving ≤100 arcseconds post-PL versus 13.64 % post-surgery, mirror outcomes in surgically aligned intermittent exotropia,^18^^,^^19^ yet exceed rates seen in non-PL esotropia cohorts (20–30 %).^20^ The correlation between PL duration and stereoacuity gains (*r* = −0.51, *P* = 0.002) underscores the dose-dependent nature of neural plasticity, consistent with fMRI studies linking PL to increased interhemispheric coherence.

Visual perceptual learning through practice or training in perceptual tasks, such as the detection of visual gratings, stimulus orientation judgment, texture discrimination, retinal location recognition, and hyperacuity and vernier tasks, can progressively promotes perceptual performance. Perceptual learning may reflect plasticity in a complex set of brain networks.^14^, ^32^, ^33^, ^34^ A number of studies have described the importance of binocular vision and supported the effectiveness of perceptual learning for intermittent exotropia,^16^, ^17^, ^18^, ^19^ and this is the first study to evaluate the effectiveness of perceptual learning for esotropia after strabismus surgery, highlighting another therapeutic approach to treating esotropia. Notably, there existed five cases (11.26 %) with consecutive exotropia at the final follow-up. The incidence of consecutive exotropia secondarily to esotropia is estimated to 4 %∼27 %.^35^^,^^36^ Early onset of esotropia, preoperative angle of distance deviation, presence of postoperative adduction deficit, amblyopia and hyperopia are the risk factors for the development of consecutive exotropia.^37^, ^38^, ^39^ Since our study lack of control groups, we cannot elucidate the relationship between perceptual learning and consecutive exotropia, and further research is needed.

This study has several limitations that should be acknowledged. First, its retrospective design and the inclusion of four distinct types of esotropia may introduce inherent biases and constrain the ability to establish causal inferences. Second, the relatively small sample size limits the statistical power and precision of our findings, potentially increasing the risk of Type II errors (false negatives) and affecting the generalizability of the results across the broader esotropia population. Heterogeneity in age, preoperative visual function, and compliance patterns further underscores the need for caution in interpreting outcomes. Despite these limitations, we wish to emphasize that the primary findings remain consistent and clinically informative. To address these constraints in future work, we strongly advocate for prospective, randomized controlled trials (RCTs) with larger cohorts. Such studies would help validate the efficacy of perceptual learning protocols, establish evidence-based guidelines for post-surgical visual rehabilitation, and optimize personalized treatment strategies for esotropia patients.

During the follow-up period, we observed suboptimal adherence to the prescribed training regimen among some pediatric participants. This lack of engagement could be attributed to a combination of factors, including a lack of intrinsic motivation towards the tasks, the perceived repetitiveness of the exercises, or a mismatch between the stimulus characteristics and the child's individual preferences and cognitive profile. These observations underscore a critical challenge in perceptual learning interventions: sustaining children's motivation and active participation over time. In response, we propose that future iterations of perceptual learning programs necessitate a fundamental optimization. This can be achieved through the integration of Artificial Intelligence (AI)-driven adaptive algorithms that dynamically personalize task difficulty and content based on real-time performance feedback, thereby maintaining an appropriate challenge level and reducing monotony. Concurrently, Virtual Reality (VR)-based immersive environments can be leveraged to transcend mere entertainment; they offer a platform to create engaging, context-rich, and rewarding experiences that align with pediatric interests. By fully harnessing the synergies between AI and VR, we can significantly enrich the training content and context, address the root causes of poor compliance and foster a more profound and sustained engagement throughout the rehabilitation process.

In conclusion, perceptual learning can improve the contrast sensitivity at medium SF (6cpd) and high SFs (12 cpd, 18 cpd) and promote the recovery of stereoscopic vision after esotropia surgery, displaying great application prospects for the treatment of esotropia. Our results challenge the conventional focus on ocular alignment as the endpoint of esotropia management. By addressing residual cortical dysfunction, PL bridges the gap between surgical success and functional vision restoration. The 86 % compliance rate highlights the feasibility of home-based digital therapy in children, overcoming scalability barriers of clinic-based regimens. Notably, PL’s benefits emerged despite heterogeneous esotropia subtypes, suggesting broad applicability across etiologies.

## Conclusion

This study establishes perceptual learning as a viable strategy to augment surgical outcomes in esotropia, targeting neural deficits refractory to alignment alone. By restoring medium-to- high-SF contrast sensitivity and stereopsis, PL empowers patients to achieve not only ocular normality but functional visual proficiency. These findings advocate for integrating PL into post-surgical protocols, heralding a paradigm shift toward comprehensive, plasticity-driven strabismus care.

## Declaration of competing interest

The authors have no financial or proprietary interest in the materials presented herein.

## Appendix Supplementary materials

Image, application 1

Image, image 2 dummy alt text
