# Supplementary material for: Home-based perceptual learning augments high-frequency contrast sensitivity and stereopsis after esotropia surgery: A retrospective cohort study
Source: J Optom. 2025 Dec 25;19(2):100589. doi: 10.1016/j.optom.2025.100589 (PMC13063269; doi:10.1016/j.optom.2025.100589)
Supplement: Supplementary file 1 [file mmc1.docx]

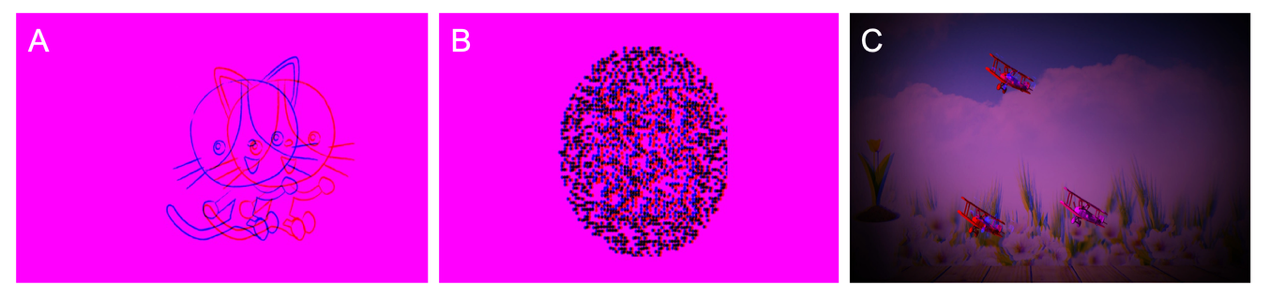


**Supplemental Figure.**​​ Examples of Doboso Fusion and Stereopsis Training Modules for Post-Surgical Perceptual Learning Tasks.

​**A. Line Fusion Task​**

Through dichoptic viewing, the left and right eyes are presented with two images that are largely identical but possess slight disparities (as shown in A, the images seen by each eye differ slightly, with control points set to prevent monocular suppression in the patient). Initially, the images seen by each eye are superimposed. As the training commences, the images gradually separate, introducing a vergence demand (either convergence or divergence). During the training, the subject must strive to fuse the images on the screen into a single percept. If diplopia (double vision) occurs, the subject presses the spacebar to restart the trial. The vergence demand can be decreased or increased using the keyboard's up and down arrow keys.

​Stimulus range: 0–20^△^ BI (base-in) for divergence / 0–30^△^ BO (base-out) for convergence.

​**B. Random Dot Fusion Task​**

Through dichoptic viewing, random-dot images are presented separately to each eye. The central target figure can only be discerned when both eyes are used simultaneously. Initially, the images for both eyes are aligned, providing zero vergence demand. The subject needs to carefully identify whether the small figure (appearing convex or concave in depth) at the center of the screen is a square or a circle. If the inner small figure and the outer large figure are both squares or both circles, press the left key; if they are different (e.g., one square, one circle), press the right key. If the central figure cannot be distinguished, press the spacebar to restart the trial. As the difficulty level increases, the vergence demand (convergence or divergence) generated by the image disparity gradually increases.

​Stimulus range: 0–15^△^ BI (divergence) / 0–25^△^ BO (convergence).

​**C. Stereopsis "Depth Sorting" Task​**

Utilizing horizontal disparity, targets are set at varying depths (near and far). During training, the subject must carefully discriminate the relative depth (distance) of several objects on the screen and click on them in sequence from nearest to farthest using the mouse. As the difficulty level increases, the number of targets increases, and the difference in horizontal disparity between the different targets gradually decreases, thereby training and refining stereoscopic acuity.

​Stereoacuity training range: 20–800 arcseconds.
