# Supplementary figures and images for: Home-based perceptual learning augments high-frequency contrast sensitivity and stereopsis after esotropia surgery: A retrospective cohort study
Source: J Optom. 2025 Dec 25;19(2):100589. doi: 10.1016/j.optom.2025.100589 (PMC13063269; doi:10.1016/j.optom.2025.100589)

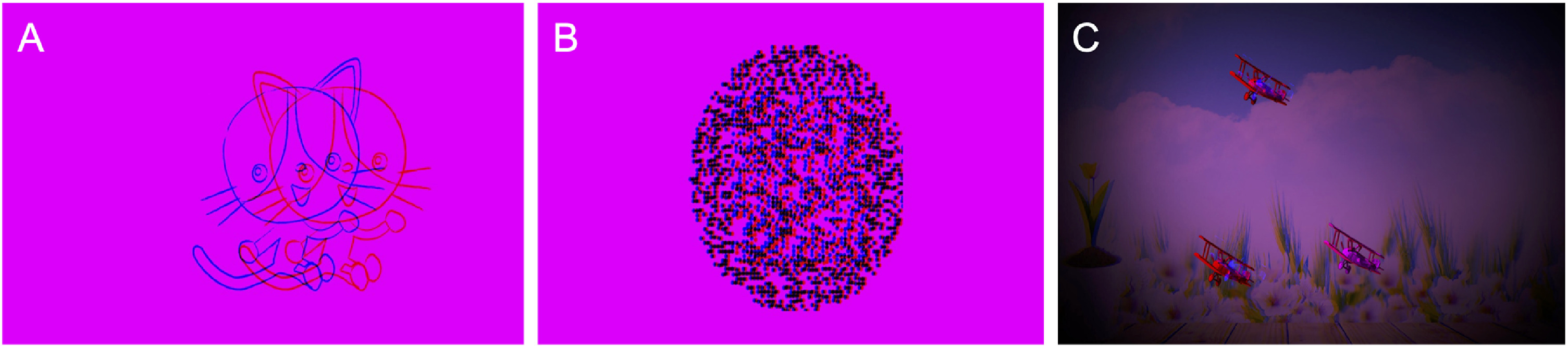

Supplement: Supplementary file 2 [file mmc2.jpg]
